# How different incentives influence reported motivation and perceptions of performance in Ghanaian community-based health planning and services zones

**DOI:** 10.1186/s13104-023-06286-2

**Published:** 2023-02-20

**Authors:** Evelyn Sakeah, Ayaga A. Bawah, Irene Kuwolamo, Maria Anyorikeya, Patrick O. Asuming, Raymond Akawire Aborigo

**Affiliations:** 1 School of Public Health, C.K. Tedam University of Technology and Applied Sciences, Navrongo, Ghana; 2 Population/Public Health Department, Navrongo Health Research Centre, Navrongo, Ghana; 3 Regional Institute for Population Studies, University of Ghana, Legon, Accra Ghana; 4 Business School, University of Ghana, Legon, Accra Ghana

**Keywords:** Community-based health delivery, Performance-based incentives, Financial and non-financial incentives, Community Health Volunteers, Maternal and Child Health

## Abstract

**Background:**

Maternal mortality is still a burden worldwide, and Ghana’s maternal and child mortalities are still high. Incentive schemes have been effective in improving health workers’ performance thereby reducing maternal and child deaths. The efficiency of public health services in most developing countries has been linked to the provision of incentives. Thus, financial packages for Community Health Volunteers (CHVs) serve as enablers for them to be focused and committed to their work. However, the poor performance of CHVs is still a challenge in health service delivery in many developing countries. Although the reasons for these persistent problems are understood, we need to find out how to implement what works in the face of political will and financial constraints. This study assesses how different incentives influence reported motivation and perceptions of performance in Community-based Health Planning and Services Program (CHPS) zones in the Upper East region.

**Methods:**

A quasi-experimental study design with post-intervention measurement was used. Performance-based interventions were implemented for 1 year in the Upper East region. The different interventions were rolled out in 55 of 120 CHPS zones. The 55 CHPS zones were randomly assigned to four groups: three groups of 14 CHPS zones with the last group containing 13 CHPS zones. Several alternative types of financial and non-financial incentives as well as their sustainability were explored. The financial incentive was a small monthly performance-based Stipend. The non-financial incentives were: Community recognition; paying for National Health Insurance Scheme (NHIS) premiums and fees for CHV, one spouse, and up to two children below 18 years, and; quarterly performance-based Awards for best-performing CHVs. The four groups represent the four different incentive schemes. We conducted 31 In-depth interviews (IDIs) and 31 Focus Group Discussions (FGDs) with health professionals and community members.

**Results:**

Community members and the CHVs wanted the stipend as the first incentive but requested that it be increased from the current level. The Community Health Officers (CHOs) prioritized the Awards over the Stipend because they felt it was too small to generate the required motivation in the CHVs. The second incentive was the National Health Insurance Scheme (NHIS) registration. Community recognition was also considered by health professionals as effective in motiving CHVs and work support inputs and CHVs training helped in improving output. The various incentives have helped increase health education and facilitated the work of the volunteers leading to increased outputs: Household visits and Antenatal Care and Postnatal Care coverage improved. The incentives have also influenced the initiative of volunteers. Work support inputs were also regarded as motivators by CHVs, but the challenges with the incentives included the size of the stipend and delays in disbursement.

**Conclusion:**

Incentives are effective in motivating CHVs to improve their performance, thereby improving access to and use of health services by community members. The Stipend, NHIS, Community recognition and Awards, and the work support inputs all appeared to be effective in improving CHVs’ performance and outcomes. Therefore, if health professionals implement these financial and non-financial incentives, it could bring a positive impact on health service delivery and use. Also, building the capacities of CHVs and providing them with the necessary inputs could improve output.

## Background

Maternal mortality is still a burden worldwide, and an estimated 211 deaths per 100,000 live births occurred in 2017 [1]. While significant progress has been made to decrease these deaths, statistics still show that the Sustainable Development Goal 3 (SDG) of achieving less than 70 maternal deaths per 100,000 live births by 2030 may not be realized [1]. Sub-Saharan African countries recorded the highest maternal mortality rates in the world, and Ghana’s maternal mortality rate was estimated at 308 deaths per 100,000 live births in 2017 [1].

In 2019 alone, an estimated 7.2 million children, adolescents, and youth died mostly from preventable or treatable causes. Of these deaths, 5.2 million occurred in the first 5 years, with almost half of these in the first month of life. Sub-Saharan Africa remains the region with the highest under-5 mortality rate in the world [2]. Even though programs have become more effective in addressing under-five mortality, the proportion of deaths occurring in the neonatal period (first 28 days after delivery) has declined marginally in recent years [3]. Although Ghana had made progress in improving maternal and child health, the country is not likely to achieve SDG 3 [1].

Several studies have revealed the important role Community Health Volunteers (CHVs) play in primary healthcare activities by improving access to health services [4–14] and maternal and child health indicators [15–20]. Conversely, the poor performance of CHVs is still a challenge in health service delivery in many developing countries [21, 22] though the reasons for these persistent problems are understood, we need to find out how to implement what works in the face of political will and financial constraints. Although insufficient training, logistics, and deficient provider knowledge are contributory factors, lack of motivation is reported to be associated with CHVs’ poor performance [22–24] Incentive schemes have been documented as effective strategies to inspire the motivation and performance of health workers in health systems in developing countries [22–24]. Incentives are designed to increase performance by boosting the value people assign to work goals, causing them to make stronger commitments to those goals and achieve them. The WHO guidelines stipulated that practicing Community Health Workers (CHWs) should be given financial packages commensurate with the job demands, complexity, number of hours worked, training, and roles that they undertake. The financial packages for CHWs serve as enablers for CHWs to be focused and committed to their work [25]. The efficiency of public health services in most developing countries has been linked to the provision of incentives [26–28] or financial packages.

Performance-based incentives (PBIs) have been implemented to transform the health system and improve maternal and child health services in many sub-Saharan countries [22, 29]. Social research has revealed how community health workers received PBIs, which contributed to improving maternal and child health indicators in Ghana [22]. According to Aninanya et al. (2016), PBIs encouraged health workers to work harder and be more punctual, increasing reported pride and job satisfaction [30]. In Zambia, the implementation of performance-based financing schemes resulted in a significant increase in job satisfaction and a decrease in attrition [31]. A study in Nigeria also revealed that PBIs helped to motivate health workers for better work performance [32]. A study on the impact of removing performance-based financial incentives on community health worker motivation in Bangladesh showed that when PBIs are removed from a CHW program, it could negatively impact CHW motivation [30]. Other studies have reported providing both financial and non-financial incentives for CHVs [31, 33], and several others have stated the outcome of these incentives on CHVs’ effective participation [22, 34–38] and improved retention [39].

However, there is growing literature suggesting that PBI is a fraught way to pay CHWs. The main concern and the growing consensus are that PBIs may appear to influence behavior, but they also open the door for “gaming” the system, which can be distracting and corrosive to overall performance. This is because PBI encouraged an uneven focus on certain activities due to their association with higher incentives, especially when CHWs had no basic remuneration, leading to the neglect of other important activities or responsibilities. Also, CHWs expressed dissatisfaction with PBI models regarding amounts paid and inconsistent and incomplete payment of incentives [25]. Research from Zambia revealed that the implementation of PBI schemes increased job satisfaction and decreased the number of CHWs who left the job but had no significant effect on motivation [28]. Bhatnagar and George emphasized that PBI strategies could succeed in motivating health workers by bringing about a change in incentives and working conditions. However, such programs need to be aligned with human resource reforms. They concluded that health workers respond to improved incentives and working conditions, but that support must be comprehensive [29].

Community Health Volunteers have been an integral part of the health system in Ghana [40–42]. These cadres of health workers have been trained to provide health services in rural communities to augment the work of health professionals [16]. Research has reported the contributions of CHVs to health programs [9, 10, 40–42]. The introduction of the Community-based Health Planning and Services (CHPS) program in rural communities strengthened the collaboration between CHVs and health professionals in providing basic services in rural communities [43]. The CHPS program was established in 2000 to increase the availability and access to basic health services [44]. A CHPS zone is a geographical coverage area for community services with a population of 3000 to 4500 people, covering two to three-unit committees of the district assembly. The overall strategic goal of CHPS is to improve the health status of the population by strengthening the health system and empowering communities and households for service delivery and utilization [45]. CHVs are non-salaried community members who are trained to support Community Health Officers (CHOs) in providing basic services in a CHPS zone [44].

## Methods

### Study setting

The Upper East region is located in the northeastern corner of Ghana, bounded by Burkina Faso to the north and the Republic of Togo to the east. It covers an area of 8,842 square kilometers. The 2010 Census put the population of the Upper East region at 1,046,545, which is predominately rural [46]. The qualitative study was carried out in eleven districts (Kassena-Nankana Municipality, Bolgatanga Municipality, Kassena-Nankana West District, Builsa North District, Builsa South District, Bongo District, Talensi District, Bawku Municipal, Bawku West District, Binduri and Garu-Tempane Districts) in the Upper East region of Ghana. At the time of initiating the CHPS + project the region had 13 districts, but due to redistricting the number of districts has increased to 15. The 11 districts were selected because they were either part of the intervention or control districts: two CHPS zones in each of the 11 districts were selected to participate in the study.

### Study design and methods

A quasi-experimental study design with post-intervention measurement was used. Performance-based interventions were implemented for 1 year in the Upper East region. A total of 55 CHPS zones received the incentive interventions. The 55 CHPS zones were randomly assigned to four groups: three groups of 14 CHPS zones with the last group containing 13 CHPS zones. The four groups represent the four different incentive schemes. Since the randomization was at the CHPS zone level, all CHVs working in the same CHPS zone received the same incentive. Several alternative types of financial and non-financial incentives as well as their sustainability were explored. The financial incentive is a small monthly performance-based per-diem. The non-financial incentives are community recognition; paying for NHIS premiums and fees for CHV, one spouse, and up to two children below 18 years, and; quarterly performance-based awards for performing best-performing CHVs. We conducted 30 In-depth interviews (IDIs) and 31 Focus Group Discussions (FGDs) with health professionals and community members.

Qualitative research approaches were used to evaluate the CHV incentives. The indicators included: Feedback on the performance of the CHVs by the CHO, Community Health Management Committees (CHMCs), CHVs, and community, and it involved conducting FGDs and IDIs with the targeted stakeholders. We randomly selected two CHPS zones from each intervention area and targeted the following group of persons: women, men, CHOs, CHMCs, and CHVs. Women and men were selected through key informants for the FGDs. CHOs working in the selected CHPS zones were eligible for IDIs. We purposively selected 3 CHOs from each of the intervention areas who have been involved in supervising the volunteers for the interview, and also purposively selected CHMCs that work in the selected CHPS zones for the FGDs. The first 10 CHVs who consented to participate in the study were invited to participate in one FGD in each of the selected zones (Table 1).

**Table 1:** Distribution of Study Respondents

Study Population	Type of Interview	Number of Interviews
**Community Members**		
Men	FGDs	10
Women	FGDs	10
Community Health Management Committees	FGDs	11
**Health Workers**		
Community Health Officers	IDIs	15
Community Health Volunteers	IDIs	15
**Total**	**IDIs-30**	**FGDs-31**

### Training and data collection

We recruited graduate research assistants from the study districts and trained them on the interview guides and the processes involved in conducting FGDs and IDIs. As part of the training, a pilot test was conducted in the non-intervention districts to assess the clarity and appropriateness of the interview guides before the commencement of actual data collection.

Data collection lasted from 1st October 2019 to 30th November 2019. The data collection process required making prior appointments with respondents before conducting the interviews. A three-member team was formed in each district for the field activities. Each district team comprised a supervisor and three interviewers each for the FGDs and IDIs. The district supervisor provided oversight responsibility during data collection. They assisted data collectors in locating sampled communities and organized FGDs and IDIs. As much as possible, the principal investigator and the co-investigators, and supervisors observed FGDs, IDIs, and interviews administered in the study districts and supervisors offered suggestions or addressed challenges when necessary.

### Qualitative data analysis

The FGDs and IDIs with community stakeholders were conducted in the local languages while those with the health providers were in English. We audio-recorded all interviews and discussions and transcribed them verbatim into English. We reviewed the transcripts thoroughly for accuracy and completeness and corrected them to facilitate coding by theme. The Principal Investigator (ES) and two other Co-Investigators (RA and IK) sorted the transcripts by sources and conducted multiple readings, writing memos in the margins of the text in the form of short phrases, ideas, or concepts arising from the texts. We used these memos to iteratively develop coding categories. Using thematic analysis, we closely examine the data to identify common themes–topics, ideas, and patterns of meaning that came up repeatedly and themes that were atypical in response to each question. Transcripts were imported into NVIVO 11.0 for open, axial, and selective coding by three separate coders (ES, RA, and IK). Coders met regularly to discuss the process of coding, revise the codebook as necessary, and resolve any uncertainty in coding. The themes were used to generate reports that allowed us to describe the thoughts and opinions within the interviewee group (e.g., community stakeholders) as well as compare responses across groups (e.g., community stakeholders and health professionals).

## Results

### Types of incentives given by KOICA-Ghana Health Service

The types of incentives given to the volunteers included Stipend, NHIS registration or renewal, Awards, and Community recognition. When asked what incentives they received, the CHVs mentioned those provided by KOICA as contained in the excerpts below.

*” Yes, the CHVs have ever received the health insurance renewal and registration incentive for themselves and their families. They always tell them to bring four people from their families for their health insurance to be renewed. Even if they do not have one, they will do a new one for them. It is done yearly and every year it is done for them.”* (IDI-CHO-Nungu Talensi)

For community members, work support inputs such as bicycles, raincoats, and wellington boots were regarded as incentives. They reported that logistics such as bicycles generally facilitated the movement of volunteers within the communities to deliver health services to the people.

*….” Truly they gave them bicycles, raincoats, wellington boots for use, especially during the rainy season; if a child fell sick at a time that it was raining and his services were needed, he would wear them and attend to duty. If it was not a minor sickness, they would be referred to the next level health facility.”* (FGD-A female participant-Kalijiisa-Builsa North)

### Ranking of incentives by community members

The community respondents generally wanted the first incentive to be the Stipend. They argued that the CHVs have needs and that the stipend could help them meet those needs. Although they thought the NHIS registration and renewal were also a huge motivation, they said that if the stipend is substantial, then the CHVs could use the money to do their own NHIS registration and renewal.

*…” As for me, I think the money will be good because if they are to wait for the whole year before the community calls them and award them, how much will they give them? How can they just fold their arms and be sitting and waiting for that award when we all know they work every day and also need to eat something so I think the money will be more effective. With the NHIS, when they get the money, they will renew it themselves.”* (FGD-A male participant-Nabango, Kassena-Nankana West)

The CHVs themselves preferred the stipend. Here is how some of the CHVs ranked the incentives.

*…” You see everything now is about money before any other thing, when you give them money, they can use it to renew their NHIS.”* (*IDI-CHV-Manga-Binduri*)

*…” I will prefer the small token given me first, and then the bicycle, raincoat, and wellington boots.* (IDI-CHV-Pindaa-Kassena-Nankana Municipal)

Those who argued for the Awards to come first were of the view that it creates competition and therefore pushes the volunteers to deliver their best to get awarded. In addition, the CHVs said the Awards raise their self-esteem as the community publicly acknowledges their efforts.

…. “*The awards should come first because they give you morale to work.”*(IDI-CHO-Nabango-Kassena-Nankana West)

…” *I think the award will come first because if at the end of every year they organize a durbar and gently hand us some awards ranking from best performing volunteer it makes the people also know our importance in the community and recognize our work.”*(IDI-CHV-Nabango)

Some of the CHOs also prioritized the Awards over the stipend because they felt that the stipend was too small to generate any significant motivation in the volunteers to improve their performance. Here is what some of them had to say;

*…” I will go for the award because if the volunteer knows that they are all competing to get it, he will put in his effort. It will even increase the number of home visits the person does because he knows that I am not the only person that is fighting for the award, other people are fighting for the award so because of that I have to do my work more to earn the award. Yes. So, I would go for the award.”*(IDI-CHO-Pumpongo-Bolga Municipal)

This view of the CHOs was supported by some members of the community as contained in the excerpt below;

….” *I think the award has an honor that would last for many years and even gives them the enthusiasm to work more. Any other incentive will not honor like the award. If for instance, the health Director comes and the community entertains them and a motorbike is given to the volunteers for his/her hard work, even if the fellow is as old as 63, he/she will still do the work.”*(FGD-A member of the CHMC-Kalijiisa-Builsa North)

Those who did not prioritize the awards over the stipend indicated that the awards were not frequent enough and only benefitted a few and so would not be motivating enough compared to the stipend.

The argument for community recognition to be first was based on the lasting effect of the incentive on the CHVs. Those who preferred the Community recognition said the following;

*I was hoping they could put community recognition first. The arrangements are not ok; sometimes some people do not need money in their lives compared to being grateful for the work they have done.*(IDI-CHO-Nungu-Talensi)

A couple of respondents also prioritized the NHIS registration over the other incentives. They said if the volunteers are not healthy, they cannot perform their duties, and therefore it was important to ensure that they are healthy at all times. They also said registration of NHIS and renewal is a major challenge for most community members including the CHVs and so, any program that provides such an incentive should be supported.

*….” For the Talensi district, I will say NHIS. It is NHIS that is effective here whereas other districts have other incentives that they receive and for that reason, I cannot tell best which incentive is more effective.”*(IDI-CHO-Nungu-Talensi)

From the excerpt above, the preferences of the respondents appeared to be influenced by the type of incentives that were implemented in their communities.

After the stipend, almost all the CHMCs were of the view that the second incentive to prioritize should be the NHIS registration. This was supported by the community members and the CHVs.

…” *I think that if the NHIS can follow the stipend because it is when you are healthy that you can work; when you have the card and you can treat yourself all the time. it will motivate you to do the work well.”*(FGD-A member of the CHMC-Soogo-Bawku West)

The CHOs wanted the Stipend second.

*….” The money aspect will come second because children and other issues the CHVs need to take care of.”*(IDI-CHO-Nabango-Kassena-Nankana West)

Apart from complaints that the stipend was small, most of the respondents also complained about delays in the disbursement.

*….” Yes, but I will say it is 50, 50 because some of the incentives come on time but for the monetary aspect, it delays a lot and I do not know if something could be done about that.”*(IDI-CHO-Kalvio-Kassena-Nankana West)

## Changes in the work attitudes of volunteers

Before the introduction of the incentives for CHVs, apathy towards work by CHVs was common and this affected their outputs. The CHVs said.

…… “*At first, they used to give us soap, mosquito nets, and insecticides. They used to call us at the District Health Administration for pieces of training and at the end of the training, they will give us some allowance and we are happy.”*(IDI-CHV-Bawku West)

However, they claimed, these are things that stopped for a long while until KOICA implemented its interventions. The CHOs who mainly benefited from the support of the CHVs in providing healthcare to the community acknowledged that complaints from CHVs about the lack of incentives in the performance of their duties were a significant factor in the provision of healthcare to the people. They reported that KOICA’s intervention tackled this critical need which ultimately led to improved services by the CHVs. The CHOs said the following:

*….” Hmmm because KOICA last provided them with bicycles, raincoats, and books and if you are comparing to when they were not having them, and today I think a lot has changed. When it even rains, they can still go out to work, when it is night because they were given torch lights which push them to do the work. Also, the bicycles they were given are used to travel to houses far away. So, I think it has improved better than the previous years. As I already told you, the logistics supplied by KOICA have made an increase in the number of home visits.”*(IDI-CHO-Nungu-Talensi)

All CHMCs acknowledged the lack of work support inputs for the work of the volunteers in the past. They said, for instance, the bicycles have facilitated their movement between compounds and therefore they can cover their communities within a short time.

*….” Yes, there is change; what they used to do for the past 2 years they used not have the logistical support we mentioned, they will want to visit the next house but no means but now with the bicycle given to them they can go anywhere in the community within the shortest possible time. First, they will want to walk but mud everywhere but now there are wellington boots, now there is a phone so what they could not do, now they can do all, then they used to sacrifice at first there was not any motorking but now we have it to take care of our women in labor for safe delivery.”*(FGD-A member of the CHMC-Amanga-Bongo)

The overwhelming majority of the CHVs confirmed that indeed, incentives were enablers because they gave them the zeal to deliver their best. It also gave dependents of the CHV hope and therefore they were more likely to encourage the volunteer to continue supporting the health system.

*….” Yes, it will encourage me to work. You know, if you say goodbye to your family that you are going to work and they are assured that you will return home with something, they will even encourage you to go to work. but if you are working and you do not return home with any benefit, your family will not encourage you to work*.” (IDI-CHV-Builsa North)

Most of CHMCs also observed increases in the outputs of the volunteers as a result of the logistics given to them.

*….” In those days, many would not bother visiting the health service providers when pregnant and would not also be delivered at the health centre: An act which is full of challenges. The children were also not taken to the centre for weighing, PNCs, and what have you. As such, the children grew to be weaklings. The education and drugs we get from the volunteers now have brought change.”*(FGD-A member of the CHMC-Bachongsa-Builsa South)

All the respondents said the volunteers now conduct their compound visits more frequently than before. Some reported that household visits have become a daily routine for the volunteers. The CHMC also reported that processes such as supervision has improved as a result of the KOICA interventions. One of those improvements has been accountability to the CHMC. The CHVs did not report to the CHMCs but this changed as a result of the KOICA interventions. The CHMCs are now able to monitor their activities and this has led to improvements in their outputs.

*….” In the past year, they used to not report to us after home visiting but the coming of KOICA has changed this attitude; they now report to us and we monitor their work every month because of that their performance has increased compared to the previous years*.” (FGD-A member of the CHMC-Pumpongo-Bolga)

They have reported that inputs such as equipment and supplies that were given to facilitate the work of the volunteers and acknowledged that the equipment and other logistics were helping them improve their services.

*….” When the items were not there, we were not able to work but now they can call you for any information and you will call them and give it to them and go round before it will get dark or sunset but it is just that all those items are faulty. First, we were working in darkness but now there is a torchlight, wellington boots, a mobile phone, and a bicycle. Now using the bicycle has helped us improve a lot because we have been called to the office several times for praise.”*(IDI-CHV-Gentiga-Bawku Municipal)

Some discussants attributed improvements in the health indicators to the work support inputs and processes the volunteers received from KOICA, and the incentives given to them.

## Improvements in health education

Volunteers were reported to have been more effective in the provision of health education in all the zones where the various incentives were implemented. Most men in the community testified that CHVs now come to call them to the CHPS compound for health talks some of which focus on hygienic practices. Through home visits, health education in communities has been effective. The CHO supported this observation.

…” *Yea, what has changed when you visit the people now in their homes, you see that the environment is clean. There is improvement in the environment. The volunteers have been providing health education in the communities and one can see that people are practicing what they hear from the volunteers and that would ultimately lead to healthy lives.”*(IDI-CHO-Dabilla-Garu)

The community members reported that since the introduction of the incentives, coverage of health interventions has increased.

*….” We appreciate the CHVs’ work in the sense that our health indicators have gone higher because we are now in better health conditions than before. We appreciate their errands to our doorstep. This makes us so appreciative of their services.”*(FGD-A female participant-Bachongsa-Builsa South)

Some of the women reported that the volunteers have improved their visits to the households. While some talked about daily visits, others said they visit monthly and that these have been more regular compared to the past. They also observed improvements in follow-ups and they attributed it to the work support inputs provided to the volunteers.

*….” Now they visit us every month and it is better than the past two years that they may not even visit you at home but for now, there is an improvement.”*(FGD-A female participant-Manga-Binduri)

All the CHOs also observed the changes.

*…. “The change is in the home visits; at first, they will tell you that they have gone for home visits but no evidence to show and the pregnant women were not coming but now that has changed”.*(IDI-CHO-Manga-Binduri)

As observed by the other discussants and interviewees, all the CHVs reported that the provision of inputs has helped to improve their services. The bicycles have facilitated their movement between compounds, helped them to trace defaulters, and eased follow-up visits to compounds. Raincoats and wellington boots have helped them to deliver health services to the people regardless of the terrain or the prevailing weather condition.

*…” At first, I used to be somehow lazy in my work because I would work all day and get nothing out of it to even support myself; how to move from one house to the other was a problem. I had to walk every day but since they gave me the logistics it encourages me to work hard. I have come to realize that it is not free as I thought because people are watching me and if I still do my best, it could even be more than what I received so far.”*(IDI-CHV-Amanga-Bongo)

Even in the control areas, where CHVs were only trained and given work support inputs such as bicycles, raincoats, and wellington boots to facilitate health service delivery, it also led to improvement of home visits and health education, and defaulter tracing in such communities.

There were reports that the KOICA CHPS + project trained the volunteers to provide various services and some community members considered that an incentive. They said that the training had broadened the range of services they provided and also honed their skills to continue to better provide other services.

*….” What I want to add is like my brother rightly said if you want to compare 2018 to that of this 2019 because 2018, they did not have enough training for them, the volunteers by then did not know how to do malaria tests but now they can do it. I mean our small nurses in the house; they did not have any means in case of emergency they could use but now they gave them bicycles for their movements. In 2019, we organize durbar and they introduced them to everyone so we know what they can do for the community.”*(FGD-A male participant-Nabango-Kassena-Nankana West)

## Discussion

This study identified the type and ranked the incentives given to CHVs and assessed the effectiveness of those incentives on work performance and their sustainability in CHPS zones. Community Health Volunteers were given incentives and included a Stipend, Awards, NHIS registration, and Community recognition. CHVs preferred the stipend and the NHIS and the Awards in that order. Logistics to facilitate service delivery were regarded as incentives by CHVs in the intervention and control areas. Motivating the CHVs has helped improve compound visitations, and CHVs accountability to CHMCs: The CHMCs are now able to monitor the activities of CHVs and this has led to improvement in their outputs. The respondents emphasized that incentives should be sustained beyond the project period and that community recognition be an integral part of the volunteer concept.

Studies have demonstrated the use of incentives to improve the job performance of community health workers [22, 26–28, 37, 47–51]. The KOICA CHPS + project rolled out four types of incentives in CHPS zones. Implementing these incentives helped to identify the most preferred incentives by CHVs. Social studies have identified financial incentives as enablers in promoting CHVs performance and their retention [22, 29, 30, 33, 38, 52, 53] may be effective in improving health service delivery in Ghana. Community recognition was seen as culturally appropriate and volunteers were happy to have their work acknowledged at grand durbars within their communities. These results are consistent with studies that found Community recognition as one of the motivators of health workers [22, 29, 30, 37, 40, 54].

National Health Insurance Scheme registration and renewal for both the CHVs and three other family members seemed to be significant to motivate CHVs in the discharge of their duties. Apart from taking away the financial pressures associated with the registration and the health costs, it contributed to the CHVs staying healthy to perform their duties. According to the community stakeholders, the awards made the volunteers popular in the community because they were usually done at durbars where everybody was present. Some communities also gave CHVs gifts when the volunteers visited them in their compounds. Several studies have reported providing non-financial incentives to volunteers and have stated the outcome of these incentives on CHV’s effective participation and increased retention [22, 29, 30, 33–36, 41–43]. Work support inputs such as bicycles, wellington boots, raincoats, bags, and torches and CHVs training were reported to have motivated CHVs work performance and output, demonstrating that if CHVs are given the required logistics and training, it will motivate them to work hard to improve output. This study corroborates other studies that reported that volunteers’ performance could be strengthened by adequate logistical support [22, 55, 56].

Community health volunteers have played a vital role in improving health indicators in Ghana and elsewhere [9–11, 15–17, 22, 29, 44, 45, 53]. Our study revealed that health education reportedly increased in the intervention areas because of the incentives. Even in the non-intervention areas, the provision of work support inputs to CHVs appeared to be an incentive and that also improved their performance in those settings. All community members reported participating in health talks. Household visits, ANC, and PNC coverage improved. The incentives facilitated the work of the volunteers and led to increased output. These findings corroborate earlier studies that revealed that financial and non-financial performance incentives could improve both the use and quality of health care [55, 56]. Thus, financial and non-financial incentives for CHVs could be seen as enablers in increasing health delivery services thereby improving maternal and child health indicators.

### Study limitations

 This study has several limitations that including recall bias. This could have limited the validity of the data because some participants could have forgotten about past events involving the various incentives to CHVs in the CHPS zones. To lessen recall bias, participants were asked to respond to questions about the incentives they knew of. This study focused on incentives for CHVs within the context of the CHPS program and might not be generalizable to other contexts because of the uniqueness of the design and implementation of the CHPS program in the Upper East region. However, our findings are similar to other programs in developing countries that involved financial and non-financial incentives for CHVs in rural communities. Respondent bias may have occurred since some respondents were direct beneficiaries of the incentives. Notwithstanding these challenges, the respondents openly discussed the subject matter and the incentives given to CHVs in the CHPS zones. Four evaluation advisors reviewed and examined the research process and data analysis to ensure that the findings are consistent and could be repeated.

## Conclusion/recommendations

Incentives are effective in motivating CHVs to improve their performance, thereby improving access to and use of services by community members. The government of Ghana could provide financial or non-financial incentives to CHVs to motivate them to improve their performance and outcome. Thus, the Stipend incentive could be given to the CHVs, if government could afford. NHIS could be negotiated with the National Health Insurance Authority to enroll all CHVs without payment of premiums and fees as is currently the case for indigents. Also, the CHV incentive policy could be considered in such a way that while having NHIS as a default option for CHV incentive, the Awards could be additionally provided in regions or districts where the funding for the Awards could be secured locally. Community recognition is an incentive that should be implemented by all because it does not cost anything to implement. Also, suppling CHVs with the required inputs and training could improve output in the long round.

## Data Availability

The data used in this study come from the improving Community-Based Primary Health Care through CHPS Strengthening (CHPS+) in the Upper East Region of Ghana project, whose authors are affiliated with the Navrongo Health Research Centre. All data generated or analyzed during this study are available in the figshare repository. https://figshare.com/s/dd009be5c265594f904f.

## Declarations

Community approval was obtained from chiefs, elders, and compound heads of the study districts before the study commenced. We also assured participants of anonymity and confidentiality before conducting the interviews.

## Abbreviations

CHPS: Community-based Health Planning and Services
CHOs: Community Health Officers
CHNs: Community Health Nurses
CHVs: Community Health Volunteers
CHMCs: Community Health Management Committees
DHMTs: District Health Management Teams
SDHMTs: Sub-District Health Management Teams
NHIS: National Health Insurance Scheme
PBIs: Performance-based incentives
SERC: Sustainable Emergency Referral Care
KOICA: Korea International Cooperation Agency
IDIs: In-depth Interviews
FGDs: Focus Group Discussions
UER: Upper East Region
PHC: Primary Health Care
ANC: Antenatal Care
PNC: Postnatal Care.

## References

[1] WHO, UNICEF, UNFPA, World Bank Group and, the United Nations Population Division. Trends in maternal mortality 2000 to 2017. 2019.

[2] UNICEF, WHO, World Bank Group, United Nations., Levels & Trends in Child Mortality. 2020. Available: https://www.unicef.org/media/79371/file/UN-IGME-child-mortality-report-2020.pdf.pdf

[3] Ghana Statistical Service (GSS), Ghana Health Service (GHS), and ICF International. Ghana Demographic and Health Survey 2014, GSS, GHS, and, International ICF. Rockville, Md, USA. 2015. Available: https://www.dhsprogram.com/pubs/pdf/FR307/FR307.pdf

[4] Bateganya M, Abdulwadud OA, Kiene SM. Home-based HIV voluntary counselling and testing (VCT) for improving uptake of HIV testing. Cochrane HIV/AIDS Group, editor. Cochrane Database Syst Rev. 2010 [cited 10 Dec 2022]. doi:10.1002/14651858.CD006493.pub4 10.1002/14651858.CD006493.pub4 PMC6464814 20614446

[5] Mdege ND, Chindove S, Ali S. The effectiveness and cost implications of task-shifting in the delivery of antiretroviral therapy to HIV-infected patients: a systematic review. Health Policy Plan. 2013;28:223–36. 10.1093/heapol/czs058 22738755

[6] Mutamba BB, Van Ginneken N, Smith Paintain L, Wandiembe S, Schellenberg D. Roles and effectiveness of lay community health workers in the prevention of mental, neurological and substance use disorders in low and middle income countries: a systematic review. MC Health Serv Res 201313412. 2013;13:412. 10.1186/1472-6963-13-412 PMC3852794 24119375

[7] Petersen I, Fairall L, Egbe CO, Bhana A. Optimizing lay counsellor services for chronic care in South Africa: a qualitative systematic review. Patient Educ Couns. 2014;95:201–10. 10.1016/j.pec.2014.02.001 24629835

[8] Hall BJ, Sou KL, Beanland R, et al. Barriers and facilitators to interventions improving retention in HIV care: a qualitative evidence meta-synthesis. AIDS Behav. 2021;21:1755–67. 10.1007/s10461-016-1537-0 PMC5332336 27582088

[9] Mushi D, Mpembeni R, Jahn A. Effectiveness of community based safe motherhood promoters in improving the utilization of obstetric care. The case of Mtwara Rural District in Tanzania. BMC Pregnancy Childbirth. 2010;10:14. 10.1186/1471-2393-10-14. 10.1186/1471-2393-10-14 PMC2858713 20359341

[10] Kuhn L, Zwarenstein M. Evaluation of a village health worker programme: the use of village health worker retained records. Int J Epidemiol. 1990;19:685–92. 10.1093/ije/19.3.685 2262265

[11] Doctor HV. Has the Navrongo Project in Northern Ghana been successful in altering fertility preferences? Afr Popul Stud. 2007;22:87–106.

[12] Kironde S, Kahirimbanyi M. Community participation in primary health care (PHC) programmes: Lessons from tuberculosis treatment delivery in South Africa. Afr Health Sci. 2002;2:16–23. PMC2141563 12789110

[13] Dudley L, Azevedo V, Grant R, Schoeman JH, Dikweni L, Maher D. Evaluation of community contribution to tuberculosis control in Cape Town, South Africa. Int J Tuberc Lung Dis Off J Int Union Tuberc Lung Dis. 2003;7:48–55. 12971654

[14] Haldane V, Chuah FLH, Srivastava A, Singh SR, Koh GCH, Seng CK, et al. Community participation in health services development, implementation, and evaluation: a systematic review of empowerment, health, community, and process outcomes. PLoS ONE. 2019;14:e0216112. 10.1371/journal.pone.0216112 PMC6510456 31075120

[15] Pangu KA. The Bamako Initiative 50. 1997;5:26–7.

[16] Hounton S, Byass P, Brahima B. Towards reduction of maternal and perinatal mortality in rural Burkina Faso: communities are not empty vessels. Glob Health Action. 2009;2. 10.3402/gha.v2i0.1947. 10.3402/gha.v2i0.1947 PMC2779943 20027267

[17] Haines A, Sanders D, Lehmann U, Rowe AK, Lawn JE, Jan S, et al. Achieving child survival goals: potential contribution of community health workers. Lancet. 2007;369:2121–31. 10.1016/S0140-6736(07)60325-0. 10.1016/S0140-6736(07)60325-0 17586307

[18] Nii Bhuinneain GM, Mccarthy FP. A systematic review of essential obstetric and newborn care capacity building in rural sub-saharan Africa. BJOG Int J Obstet Gynaecol. 2015;122:174–82. 10.1111/1471-0528.13218 25546037

[19] Okwundu CI, Nagpal S, Musekiwa A, Sinclair D. Home- or community-based programmes for treating malaria.Cochrane Database Syst Rev. 2013. 10.1002/14651858.CD009527.pub2 PMC6532579 23728693

[20] Peter Adatara JA. Challenges of being a hospital nurse manager in the Volta region of Ghana: a qualitative study. Nusing Manag Harrows. 2019;25:35–42. 10.7748/nm.2018.e1773 30479101

[21] Boakye MDS, Owek CJ, Oluoch E et al. Challenges of achieving sustainable community health services for community case management of malaria. BMC Public Health. 2018;18. Available: 10.1186/s12889-018-6040-2 10.1186/s12889-018-6040-2 PMC6167894 30285684

[22] Sakeah E, McCloskey L, Bernstein J, Yeboah-Antwi K, Mills S, Doctor HV. Is there any role for community involvement in the community-based health planning and services skilled delivery program in rural Ghana? BMC Health Serv Res. 2014;14:340. 10.1186/1472-6963-14-340. 10.1186/1472-6963-14-340 PMC4251607 25113017

[23] Kweku M, Manu E, Amu H, et al. Volunteer responsibilities, motivations and challenges in implementation of the community-based health planning and services (CHPS) initiative in Ghana: qualitative evidence from two systems learning districts of the CHPS + project. BMC Health Serv Res. 2020;20:482. 10.1186/s12913-020-05348-6 PMC7260774 32471429

[24] WHO. WHO guideline on health policy and system support to optimize community health worker programmes. 2018. Available: https://www.who.int/publications/i/item/9789241550369 30431747

[25] WHO. WHO guideline on health policy and system support to optimize community health worker programmes. 2018 Aug. Available: https://www.who.int/publications/i/item/9789241550369 30431747

[26] Akazili J, Adjuik M, Jehu-Appiah C, Zere E. Using data envelopment analysis to measure the extent of technical efficiency of public health centres in Ghana. BMC Int Health Hum Rights. 2008;8:11. 10.1186/1472-698X-8-11. 10.1186/1472-698X-8-11 PMC2605432 19021906

[27] Aninanya GA, Howard N, Williams JE, Apam B, Prytherch H, Loukanova S, Kamara EK, Otupiri E. Can performance-based incentives improve motivation of nurses and midwives in primary facilities in northern Ghana? A quasi-experimental study.Glob Health Action.2016;9. 10.3402/gha.v9.32404 PMC5065697 27741956

[28] Shen GC, Nguyen HTH, Das A et al. Incentives to change: effects of performance-based financing on health workers in Zambia. Hum Resour Health. 2017;15. Available: 10.1186/s12960-017-0179-2 10.1186/s12960-017-0179-2 PMC5331731 28245877

[29] Bhatnagar Aarushi GS, Asha. Motivating health workers up to a limit: partial effects of performance-based financing on working environments in Nigeria. Health Policy Plan. 2016;31:868–77. 10.1093/heapol/czw002 26946273

[30] Glenn J, Moucheraud C, Payán DD et al. What is the impact of removing performance-based financial incentives on community health worker motivation? A qualitative study from an infant and young child feeding program in Bangladesh. BMC Health Serv Res. 2021;21. Available: 10.1186/s12913-021-06996-y 10.1186/s12913-021-06996-y PMC8447563 34535147

[31] Schwarz D, Sharma R, Bashyal C, Schwarz R, Baruwal A, Karelas G, Basnet B, Khadka N, Brady J, Silver Z, Mukherjee J, Andrews J, Maru SRD. Strengthening Nepal’s Female Community Health Volunteer network: a qualitative study of experiences at two years. BMC Health Serv Res. 2014;14:473. 10.1186/1472-6963-14-473 PMC4282192 25301105

[32] Greenspan JA, McMahon SA, Chebet JJ et al. Sources of community health worker motivation: a qualitative study in Morogoro Region, Tanzania. Hum Resour Health. 2013;11. Available: 10.1186/1478-4491-11-52 10.1186/1478-4491-11-52 PMC3852396 24112292

[33] Greenspan JA, McMahon SA, Chebet JJ, Mpunga M, Urassa DP, Winch PJ. Sources of community health worker motivation: a qualitative study in Morogoro Region, Tanzania. Hum Resour Health. 2013;11:52. 10.1186/1478-4491-11-52 PMC3852396 24112292

[34] Muula AS, Hofman J, Cumberland M. What motivates community health volunteers in Mecanhelas district, Mozambique? Report from a qualitative study. Ghana Med J. 2006;38:24–7. 10.4314/gmj.v38i1.35991.

[35] Dil Y, Strachan D, Cairncross S, Korkor AS, Hill Z. Motivations and challenges of community-based surveillance volunteers in the northern region of Ghana. J Community Health. 2012;37:1192–8. 10.1007/s10900-012-9569-5 22614535

[36] Mpembeni RN, Bhatnagar A, LeFevre A, Chitama D, Urassa DP, Kilewo C, Mdee RM, Semu H, Winch PJ, Killewo J, Baqui AH, George A. Motivation and satisfaction among community health workers in Morogoro Region, Tanzania: nuanced needs and varied ambitions. Hum Resour Health. 2015;13:44. 10.1186/s12960-015-0035-1 PMC4458000 26044146

[37] Kironde S, Klaasen S. What motivates lay volunteers in high burden but resource-limited tuberculosis control programmes? Perceptions from the Northern Cape province, South Africa. Int J Tuberc Lung Dis. 2002;6:104–10. 11931408

[38] Luoma M. Increasing the motivation of health care workers. The Capacity Project. Tech Brief. 2006;7. Available: http://www.intrahealth.org/files/media/increasing-the-motivation-of-health-care-workers/techbrief_7.pdf

[39] Bhattacharyya K, Winch P, LeBan K, Tien M. Community health worker incentives and disincentives: how they affect motivation, retention and sustainability. Arlington, Virginia, BASICS/USAID; 2001.

[40] Cole-King S, Gordon G, Lovel H. Evaluation of primary health care–a case study of Ghana’s rural health care system. J Trop Med Hyg. 1979;82:214–28. 529346

[41] Debpuur C, Phillips JF, Jackson EF, Nazzar A, Ngom P, Binka FN. The impact of the Navrongo Project on contraceptive knowledge and use, reproductive preferences, and fertility. Stud Fam Plann. 2002;33:141–64. 10.1111/j.1728-4465.2002.00141.x 12132635

[42] Zakus JDL, Lysack CL. Revisiting Community participation. Health Policy Plan. 1998;13:1–12. 10.1093/heapol/13.1.1 10178181

[43] Ross AB, DeStigter KK, Rielly M, Souza S, Morey GE, Nelson M, et al. A low-cost Ultrasound Program leads to increased Antenatal Clinic visits and attended deliveries at a Health Care Clinic in Rural Uganda. PLoS ONE. 2013;8. 10.1371/journal.pone.0078450. 10.1371/journal.pone.0078450 PMC3813603 24205234

[44] Nyonator FK, Awoonor-Williams JK, Phillips JF, Jones TC, Miller RA. The Ghana Community-based Health Planning and Services Initiative for scaling up service delivery innovation. Health Policy Plan. 2005;20:25–34. 10.1093/heapol/czi003. 10.1093/heapol/czi003 15689427

[45] Ghana Health Service. Community-Based Health Planning and Services (CHPS)-Operational policy document. Policy Document No. 20. Ghana. ; 2005. Available: http://www.moh.gov.gh/wp-content/uploads/2016/02/CHPS-Operational-Policy-2005.pdf

[46] Ghana Statistical Service. 2010 Population and Housing Census: District Analysis Report, Bawku Municipality. Accra, Ghana; 2014. Available: http://www.statsghana.gov.gh/docfiles/2010_District_Report/Upper%20East/Bawku%20Municipality.pdf

[47] Gadsden T, Mabunda SA, Palagyi A, Maharani A, Sujarwoto S, Baddeley M, Jan S. Performance-based incentives and community health workers’ outputs, a systematic review. Bull World Health Organ Bull World Health Organ. 2021;1:11. 10.2471/BLT.20.285218. 10.2471/BLT.20.285218 PMC8542270 34737473

[48] Tripathy JP, Goel S, Kumar AM. Measuring and understanding motivation among community health workers in rural health facilities in India-a mixed method study. BMC Health Serv Res. 2016;16. 10.1186/s12913-016-1614-0. 10.1186/s12913-016-1614-0 PMC4977615 27507034

[49] Ballard M, Westgate C, Alban R, Choudhury N, Adamjee R, Schwarz R, Bishop J. McLaughlin. Compensation models for community health workers: comparison of legal frameworks across five countries. J Glob Health. 2021;11. 10.7189/jogh.11.04010. 10.7189/jogh.11.04010 PMC7916445 33692894

[50] WHO. Community Health Workers: What do you know about them? The State of evidence on programmes, activities, costs and impact on health outcomes of using community health workers. Evidence and Information for Policy. Geneva, WHO., ; 2007. Available: http://www.who.int/hrh/documents/community_health_workers.pdf

[51] WHO. Performance incentives for health in high-income countries key issues and lessons learned: World Health Report Background Paper, No 32. Geneva, Switzerland; 2010.

[52] Singh D, Negin J, Otim M, Orach CG, Cumming R. The effect of payment and incentives on motivation and focus of community health workers: five case studies from low- and middle-income countries. Hum Resour Health. 2015;13:58. 10.1186/s12960-015-0051-1 PMC4501095 26169179

[53] Kok C, Maryse D, Marjolein T, Mariam, Broerse EW, Jacqueline, Kane S, Sumit O, Hermen TM, De Mandy AM. Korrie. Which intervention design factors influence the performance of community health workers in low- and middle-income countries? A systematic review. Health Policy Plan. 2015;30: Pages 1207–1227. 10.1093/heapol/czu126 PMC4597042 25500559

[54] Bilal-Almomani A, Al-Omari. Nizar Al-momani,Mohammed Omar. The impact of incentives on the performance of employees in public sector: Case study in Ministry of labor.Eur J Bus Manag. 2017;9.

[55] Woldie M, Feyissa GT, Admasu B, Hassen K, Mitchell K, Mayhew S, McKee M, Balabanova D. Community health volunteers could help improve access to and use of essential health services by communities in LMICs: an umbrella review. Health Policy Plan. 2018;33:1128–43. 10.1093/heapol/czy094. 10.1093/heapol/czy094 PMC6415721 30590543

[56] Chatio S, Welaga P, Tabong PT, Akweongo P. Factors influencing performance of community-based health volunteers’ activities in the Kassena-Nankana Districts of Northern Ghana. PLoS One. 2019;14. doi:0.1371/journal.pone.0212166. 10.1371/journal.pone.0212166 PMC6382126 30785936

